# Regulation of Action Potential Waveforms by Axonal GABA_A_ Receptors in Cortical Pyramidal Neurons

**DOI:** 10.1371/journal.pone.0100968

**Published:** 2014-06-27

**Authors:** Yang Xia, Yuan Zhao, Mingpo Yang, Shaoqun Zeng, Yousheng Shu

**Affiliations:** 1 Institute of Neuroscience, State Key Laboratory of Neuroscience, Shanghai Institutes for Biological Sciences, Chinese Academy of Sciences, Shanghai, P. R. China; 2 Britton Chance Center for Biomedical Photonics, Wuhan National Laboratory for Optoelectronics-Huazhong University of Science and Technology, Wuhan, P. R. China; 3 State Key Laboratory of Cognitive Neuroscience and Learning & IDG/McGovern Institute for Brain Research, Center for Collaboration and Innovation in Brain and Learning Sciences, Beijing Normal University, Beijing, P. R. China; The Research Center of Neurobiology-Neurophysiology of Marseille, France

## Abstract

GABA_A_ receptors distributed in somatodendritic compartments play critical roles in regulating neuronal activities, including spike timing and firing pattern; however, the properties and functions of GABA_A_ receptors at the axon are still poorly understood. By recording from the cut end (bleb) of the main axon trunk of layer –5 pyramidal neurons in prefrontal cortical slices, we found that currents evoked by GABA iontophoresis could be blocked by picrotoxin, indicating the expression of GABA_A_ receptors in axons. Stationary noise analysis revealed that single-channel properties of axonal GABA_A_ receptors were similar to those of somatic receptors. Perforated patch recording with gramicidin revealed that the reversal potential of the GABA response was more negative than the resting membrane potential at the axon trunk, suggesting that GABA may hyperpolarize the axonal membrane potential. Further experiments demonstrated that the activation of axonal GABA_A_ receptors regulated the amplitude and duration of action potentials (APs) and decreased the AP-induced Ca^2+^ transients at the axon. Together, our results indicate that the waveform of axonal APs and the downstream Ca^2+^ signals are modulated by axonal GABA_A_ receptors.

## Introduction

In general, the dendrites and cell body receive and summate synaptic inputs, whereas the axon is responsible for action potential (AP) initiation and propagation. The axon usually functions as a reliable cable conducting APs in all-or-none (digital) mode; however, this long-held view of the axon has recently been challenged Emerging evidences has shown that subthreshold changes in presynaptic membrane potential (*V*_m_) can regulate the amplitude of AP-triggered postsynaptic responses, indicating that neurons also communicate in an analog mode [1], [2], [3]. This mode of neuronal communication may result from activities of axonal ion channels [4], [5] and receptors [6], [7] that regulate AP waveforms, presynaptic Ca^2+^ signals and, thus, neurotransmitter release [8], [9].

Ionotropic GABA_A_ receptors are one of the most important components involved in the operation of neural circuits in the central nervous system (CNS) [10]. These ligand-gated receptors are heteropentamers selectively permeable to Cl^−^ (predominantly) and HCO_3_^−^ [11]. Previous studies demonstrated that somatodendritic GABA_A_ receptors could regulate neuronal excitability, spike timing and firing pattern [12], [13]. Also there were several lines of evidence implicating a role for axonal GABA_A_ receptors in regulating neuronal activities [14], [15].

During development of the vertebrate brain, the effect of GABA_A_ receptor activation on *V*_m_ switches from depolarization to characteristic hyperpolarization. This transition is attributable to a decrease in the concentration of intracellular Cl^−^ ([Cl^−^]_i_), resulting from changes in the expression level of cation–chloride co-transporters such as NKCCs (Na^+^-K^+^-2Cl^−^ co-transporters) and KCCs (K^+^-2Cl^−^ co-transporters) [16]. It has been reported that interneurons exert a hyperpolarizing action along the entire somatodendritic axis and the axon initial segment (AIS) of CA1 hippocampal pyramidal cells [17]. However, GABA release from axo-axonic synapses onto the AIS depolarizes cortical pyramidal cells [15]. In addition, GABA_A_ receptors on hippocampal mossy fibers could depolarize presynaptic boutons, enhance neurotransmission and facilitate LTP induction [18]. Another study in cerebellar granule cells also showed an increase in spike reliability and release probability after activation of axonal GABA_A_ receptors [19]. In these studies, noninvasive approaches such as extracellular recording, cell-attached or gramicidin perforated patch recording were employed to maintain the intracellular milieu and reveal the actual function of GABA_A_ receptors under physiological conditions.

In this study, we performed direct whole-cell recording from axon blebs of layer –5 pyramidal cells to investigate the role of GABA_A_ receptors located at the main axon trunk in regulating neuronal signaling. Using gramicidin perforated patch recording from axonal blebs (>100 µm away from the soma), we found that the reversal potential of the GABA-mediated response was more negative than the resting *V*_m_ of the axon, suggesting that the activation of axonal GABA_A_ receptors could cause hyperpolarization in distal axons. In addition, we show that the GABA-induced shunting and hyperpolarizing effect can shape AP waveforms and thereby AP-triggered Ca^2+^ signals at the axon.

## Materials and Methods

### Animals

The care and use of animals complied with the guidelines of the Animal Advisory Committee at the Shanghai Institutes for Biological Sciences. This committee also approved this research. All efforts were made to minimize animal suffering and reduce the number of animals used.

### Slice Preparation

Coronal slices from prefrontal cortex were prepared from Sprague-Dawley rats (postnatal day 15–22, male). After sodium pentobarbital (30 mg/kg, i.p.) anesthesia, the animal was euthanized by decapitation. The brain was then dissected out and immersed in the following slicing solution. Slices (350–400 µm in thickness) were cut on a vibroslicer (Leica Instruments) in ice-cold slicing solution containing (in mM): 2.5 KCl, 1.25 NaH_2_PO_4_, 26 NaHCO_3_, 2 MgSO_4_, 2 CaCl_2_, 25 dextrose, 213 sucrose (315–325 mOsm, pH 7.2–7.3). After slicing, individual slices were then transferred to an incubation chamber filled with normal ACSF similar to the slicing solution but with sucrose replaced by 126 mM NaCl. Slices were then maintained in this chamber at 34.5°C for approximately 1 hour before use. For electrophysiological experiments, slices were placed in a recording chamber and perfused with the normal ACSF (36–36.5°C). All solutions were oxygenated with carbogen gas (95% O_2_ and 5% CO_2_). Cortical neurons were visualized with an upright infrared differential interference contrast (IR-DIC) microscope (BX51WI; Olympus).

### Electrophysiological Recordings

Whole-cell recordings were obtained from both the somata and the axonal blebs of layer –5 regular-spiking pyramidal neurons in prefrontal cortical slices [2], [5]. The patch pipettes had open-tip resistances of 3–6 MΩ for somatic and 8–12 MΩ for axonal recordings. In this study, we used five types of intracellular solution (ICS) for different experiments (see below). The low-Cl^−^ intracellular solution (low-Cl^−^ ICS) contained (in mM): 140 K gluconate, 3 KCl, 2 MgCl_2_, 2 Na_2_ATP, 10 HEPES, and 0.2 EGTA (280–290 mOsm, pH 7.2 adjusted with KOH). Alexa Fluor 488 (100 µM) and Biocytin (0.2%) were also added to the ICS for visualizing the cell morphology during and after recording. The low-Cl^−^ ICS was used in most of our experiments unless otherwise stated. The resistance of sharp electrodes for GABA iontophoresis was 30–50 MΩ when filled with 500 mM GABA (pH 3.6). During the experiments, a retention current (−10 nA) was applied to prevent passive leakage of GABA. Currents were sampled at 20 kHz and low-pass filtered at 3 kHz. AP waveforms recorded in current-clamp mode were sampled at 50 kHz and low-pass filtered at 30 kHz.

To exclude the contribution of somatodendritic GABA receptors, we performed recordings from axon blebs isolated from the soma. Isolated axon blebs could be obtained by sweeping a sharp electrode at the border between layer –6and the white matter [20]. We then recorded axonal blebs in the white-matter side. Data collected from blebs that were disconnected from the soma but attached with segments of the axon (usually tens of micrometers) were chosen for analysis. Similarly, Alexa Fluor 488 and Biocytin were added to the ICS to confirm that the blebs were indeed isolated from the soma. CsCl-based ICS containing 2 mM TEA and 145 mM CsCl (replacing K gluconate and KCl in the low-Cl^−^ ICS) were used in this experiment.

Patch pipette solution for gramicidin perforated patch recording contained (in mM) 140 KCl, 10 NaCl, 10 HEPES (pH = 7.2 adjusted with KOH). Gramicidin was first dissolved in DMSO to prepare a stock solution and then diluted to a final concentration of 10 µg ml^−1^ (soma) or 100 µg ml^−1^ (bleb) in the pipette solution. The gramicidin-containing solution was prepared and sonicated immediately before the experiment. To facilitate the formation of a tight seal, the tip of the pipette was dipped into and filled with a gramicidin-free solution, and then the patch pipette was backfilled with a gramicidin-containing solution. After 10–20 minutes in the cell-attached configuration, the series resistance decreased and stabilized at 100–200 MΩ (soma) or 150–300 MΩ (bleb). The series resistance was monitored during all recording sessions. Alexa Fluor 488 was also added to this pipette solution for monitoring the integrity of perforated recording. The recording was terminated upon rupture of the patch membrane, as indicated by the presence of fluorescence in the recorded cell, a sudden decrease in series resistance or a dramatic change in reversal potential.

In experiments using extracellular stimulation to evoke APs, we placed a concentric electrode at layer 2/3 near the recorded cell and delivered single electrical shocks (0.1–0.3 ms in duration) to the slice. Low-Cl^−^ ICS, modified -ICS and high-Cl^−^ ICS were used in these experiments. In the modified -ICS, the concentration of K gluconate and KCl were adjusted to 127 and 16 mM, respectively. For high-Cl^−^ ICS, they were adjusted to 72 and 71 mM, respectively.

We employed a MultiClamp 700B amplifier (Molecular Devices) for patch-clamp recording and Spike2 software (Cambridge Electronic Design) for data acquisition. The series resistance and capacitance were compensated before and after every experimental protocol. AxoClamp 900A (Molecular Devices) was used for GABA iontophoresis (200 nA, 2–5 ms) and Picospritzer III (Parker Hannifin Corporation) was used for local puffing of baclofen (10–30 psi, 15–20 ms). The *V*_m_ shown in the text and figures were not corrected with a liquid junction potential unless otherwise stated.

GABA, Picrotoxin (PTX), baclofen, CGP 35348, muscimol and Biocytin were obtained from Tocris; gramicidin from Sigma; Alexa Fluor 488, Alexa Fluor 594 and OGB-1 from Invitrogen.

### Stationary noise analysis

The membrane currents recorded in voltage-clamp mode were sampled at 20 kHz and low-pass filtered at 6 kHz; then, the current traces were used for noise analysis. For stationary noise analysis, currents were first low-pass filtered at 400 Hz using FIR digital filtering and then transformed into AC signals using DC remove with a time constant of 1 s (Spike2 software). From each recording, we chose two segments (20–40 s) of the current trace before and during drug application.

The variance of membrane current 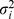 was calculated from the AC signals using the following formula:
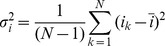
where N is the total number of points in each data segments, 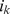 is the individual data point and 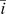 is the mean value of the current. Subtraction of the variance under control condition from those obtained during drug application produced the drug-induced variance. The average conductance of a single channel 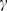 can be calculated from equation 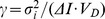
_,_ where 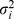 is the drug-induced variance, 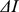 is the drug-induced change of the membrane current and 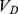 is the driving force.

Power spectra of current fluctuations were calculated as the average of fast Fourier transform of subsamples of the membrane current. Each subsample lasted for 2 s. Subtraction of baseline values from those obtained during drug application yielded the power spectra of drug-induced fluctuations. In most cases, the drug-induced spectra could be fitted by a double Lorentzian equation of the form
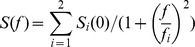
where 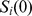 is the zero frequency asymptote value and 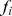 is the cut-off frequency of each component. The corresponding time constant 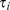 is derived from 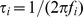. More details can be found in previous reports [21], [22].

In the experiments for stationary noise analysis, in addition to using the CsCl-based ICS described above, we also added 3 mM 4-AP, 100 µM DL-AP5, 20 µM CNQX, 100 µM CGP 35348, 100 µM CdCl_2_ and 1 µM TTX to the bath solution to minimize the baseline noise and contribution from receptors other than GABA_A_ receptors.

### Two-photon laser-scanning microscopy

Two-photon imaging was conducted on a custom-built random scanning two-photon microscope [23]. An 840-nm femtosecond laser beam (pulse width of approximately 140 fs and repetitive rate of 80 MHz) was generated by a mode-locked Ti:sapphire laser (Chameleon Vision II, Coherent) and directed into a custom-made scanner head. A pair of perpendicular acousto-optic deflectors (AODs, DTSXY-400, AA optoelectronic, France) was employed for random-access scanning. The laser beam was then relayed through a dichroic mirror (DM665) into an upright microscope (BX61WI, Olympus) equipped with a 40
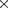
water-immersion objective. The average laser power on the sample was generally 10–20 mW. Emitted green and red fluorescence were simultaneously collected by photomultiplier tubes (H7422-40, Hamamatsu). Imaging was controlled by a LabVIEW-based program on a PC with a PCI-6259 card (National Instruments, NI, USA) for communications and a PCI-6111 card (NI) for data acquisition.

For two-photon imaging, neurons were patched with the normal ICS without EGTA but with 200 µM Oregon Green Bapta-1 (OGB-1) and 50 µM Alexa Fluor 594. Ca^2+^ transients induced by APs were monitored at an acquisition rate of approximately 1 kHz in most cases, and represented as average (10 traces, 100-points moving average) time courses of ΔF/F. To synchronize the imaging and patch clamp recording, a 5-V TTL pulse was sent from the MultiClamp 700B amplifier to the PCI-6259 card.

To examine the role of axonal GABA_A_ receptors in regulating AP-induced Ca^2+^ transients, GABA was applied through iontophoresis using sharp electrodes. For visualizing the pipette tip and estimating the distance between the tip and the axon trunk, we also added 50 µM Alexa Fluor 594 to the pipette solution.

### DAB staining

After recording, slices were transferred to 4% paraformaldehyde (PFA) in 0.1 M PB and kept in this fixative overnight. DAB staining was performed for visualization of the morphology of recorded neurons and measurement of the axon length.

### Statistical analysis

Values are presented as the mean ± s.e.m. Excel (Microsoft) and OriginPro 8 (OriginLab Corporation) were used for statistical analysis. Student’s t-test was used for statistic examination. Differences were considered to be statistically significant with P<0.05.

## Results

### GABA_A_ receptors are located in axon blebs and trunks

**Figure 1:**
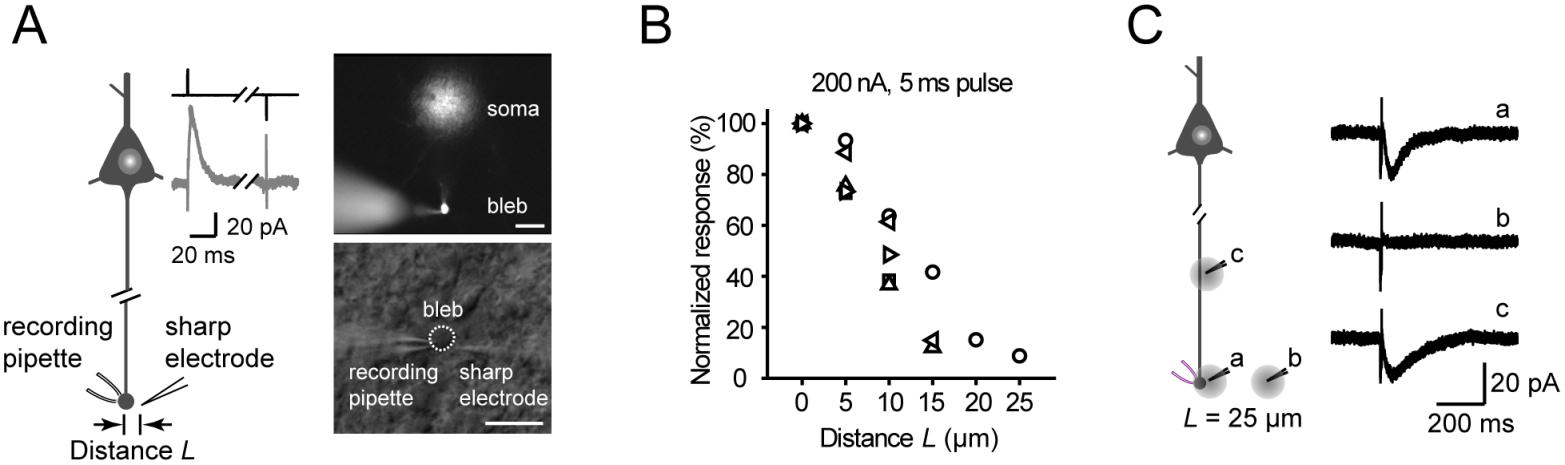
GABA receptors are located at axon bleb and trunk. A, Left, schematic diagram of bleb recording and GABA iontophoresis in a pyramidal neuron. Positive (but not negative) pulses could induce current responses. V_hold_ = –50 mV; iontophoresis pulses: 200 nA, 5 ms; retention current: –10 nA. Right, whole-cell recording from an axon bleb (top, fluorescence image; bottom, DIC image). Scale bar: 20 µm. The sharp electrode was used for GABA iontophoresis. Alexa Fluor 488 was added to the patch pipette solution so that the recording pipette was visible. B, Plot of the normalized GABA response as a function of the distance between the bleb and the tip of the iontophoresis electrode. Different symbols indicate different cells. The measurement of distance *L* is shown in the schematic diagram in panel A (indicated by arrows). C, GABA-induced responses could be observed when GABA was applied to the bleb (site *a*) or the main axon trunk (site *c*). The distance between sites *a* and *c* was approximately 50 µm, whereas that between *a* and *b* was approximately 25 µm. V_hold_ = –80 mV; iontophoresis pulses: 200 nA, 5 ms.

Previous studies have revealed the presence of GABA receptors at specific locations of the axon, including presynaptic terminals and the AIS [15], [24]. To examine whether these receptors are also expressed at the main axon trunk of layer –5 pyramidal neurons in prefrontal cortex, we performed whole-cell recordings from axon blebs, the resealed cut ends of axons formed during slicing, and applied GABA locally to the axon via iontophoresis using sharp electrodes (Figure 1A).

Because GABA is positively charged at pH 3.6, we applied a retention current of –10 nA to prevent passive leakage of GABA but extruded GABA by delivering positive current pulses (200 nA, 2–5 ms in duration). These pulses could evoke an outward current at the axon bleb recorded with a low-Cl^−^ pipette solution (7 mM [Cl^−^]_i_, holding potential: –50 mV). In contrast, no response could be observed when we applied negative current pulses (Figure 1A, inset). We next measured the diffusion distance of GABA by placing the iontophoresis electrode lateral to the recorded axon bleb with varying distance. The peak amplitude of GABA-induced currents (I_GABA_) decreased progressively with increasing distance between the bleb and the tip of iontophoresis electrode. For 5-ms pulses (200 nA), GABA responses could hardly be detected if the distance was greater than 25 µm (Figure 1B). The retention current (–10 nA) applied to the iontophoresis electrode and the limited diffusion distance suggested that GABA did not spread widely and therefore did not activate dendritic receptors. However, GABA responses could be reliably obtained if the iontophoresis was performed near the axon trunk. As shown in Figure 1C, application of GABA at a lateral site *b* that was 25 µm away from the bleb could not induce any response; in contrast, at the site *c* (approximately 50 µm away from the bleb) we could observe GABA responses similar to those at the recorded bleb (site *a*). These results indicate the presence of GABA receptors at both the bleb and the main axon trunk. The filtering effect of the axon cable could be observed when we performed whole-cell recordings at the somata and applied GABA to the axon blebs with various distance from the somata (Figure S1). As expected, the rising slope of GABA-induced currents decreased but the decay time course increased with increasing distance between the soma and the axon bleb.

**Figure 2:**
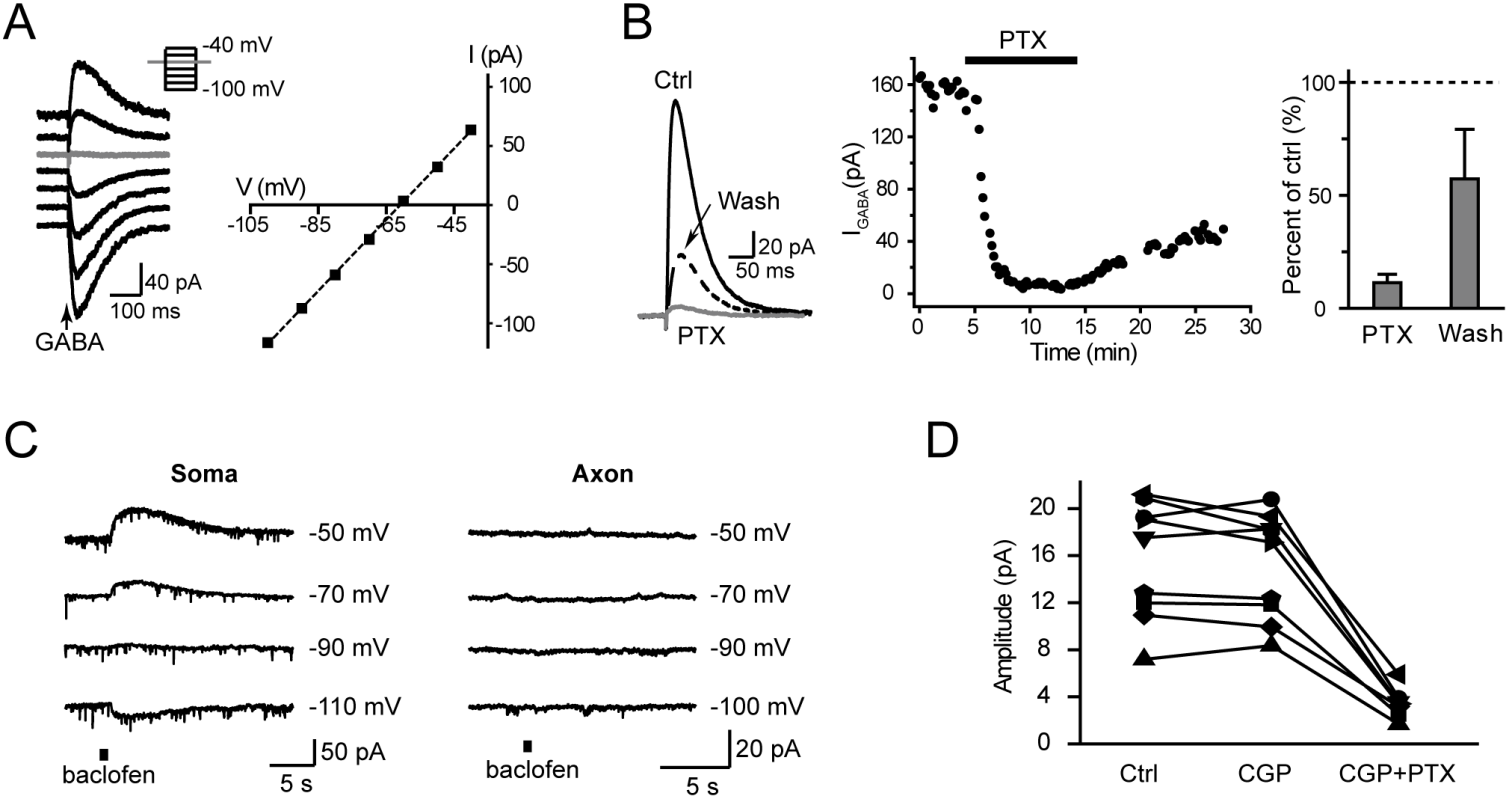
The presence of GABA_A_ (but likely not GABA_B_) receptors in the axon. A, Reversal potential of GABA responses (I_GABA_) in the axon bleb. Left, representative currents induced by GABA application at different holding potentials (from –100 to –40 mV). At –60 mV (near reversal potential), GABA application induced no obvious change in baseline current (gray). Right, I-V curve of the GABA-induced responses shown on the left. B, I_GABA_ could be blocked by GABA_A_ receptor blocker PTX. Left, example traces before (black), during (gray) and after (Wash, dashed line) the bath application of PTX (25 µM). V_hold_ = –50 mV, GABA was applied via iontophoresis. Middle, time course of the effect of PTX. Right, group data showing the change of I_GABA_ during (n = 6) and after (n = 3) PTX application. The dashed line indicates 100% of control. C, Left, currents evoked by puffing baclofen (200 µM), a GABA_B_ receptor agonist, to the soma (16 psi, 15 ms). Right, no response was observed when baclofen was applied to the axon trunk (16 psi, 20 ms). D, Group data showing that GABA-induced currents at the axon blebs could not be blocked by the GABA_B_ receptor antagonist CGP 35348 (100 µM); however, PTX could diminish these responses. Different symbols indicate different cells.

Application of GABA can activate both GABA_A_ and GABA_B_ receptors. Activation of GABA_A_ receptors mainly induces a Cl^−^ conductance, whereas activation of GABA_B_ receptors induces a K^+^ conductance. To examine the contribution of these receptors to axonal GABA responses, we measured the reversal potential of GABA-induced currents (E_GABA_) by holding the *V*_m_ at different levels (–100 to –40 mV, Figure 2A). The average reversal potential was –78.2±0.8 mV (n = 12, corrected with a liquid junction potential of 15.4 mV), similar to the calculated equilibrium potential of Cl^−^ (–78.3 mV) using the Nernst equation. Consistent with these results, bath application of 25 µM picrotoxin (PTX), a GABA_A_ receptor antagonist, could abolish the GABA-induced current recorded at the axon bleb (reduced to 11.3±3.7% of control, n = 6, Figure 2B). These results indicate that GABA responses at the axon are mediated by GABA_A_ receptors.

We next applied baclofen, a GABA_B_ receptor agonist, to further address whether GABA_B_ receptors were expressed at the axon trunk. Consistent with previous findings [25], local puff application of 200 µM baclofen at the soma induced a current with a reversal potential of –102.1±1.1 mV (n = 13, corrected with a liquid junction potential of 15.4 mV. Figure 2C), which was close to the calculated equilibrium potential of K^+^ (–107.8 mV). However, we observed no significant changes in the baseline currents (holding potential: –100 to –50 mV) after the application of baclofen to the axon bleb (Figure 2C). Consistent with these results, the GABA_A_ receptor antagonist PTX but not the GABA_B_ receptor antagonist CGP 35348 could block the currents evoked by iontophoresis of GABA at the axon (Figure 2D). Together, these results show that GABA_A_ but likely not GABA_B_ receptors (at least not GABA_B_-coupled potassium channels) are expressed along the axon.

**Figure 3:**
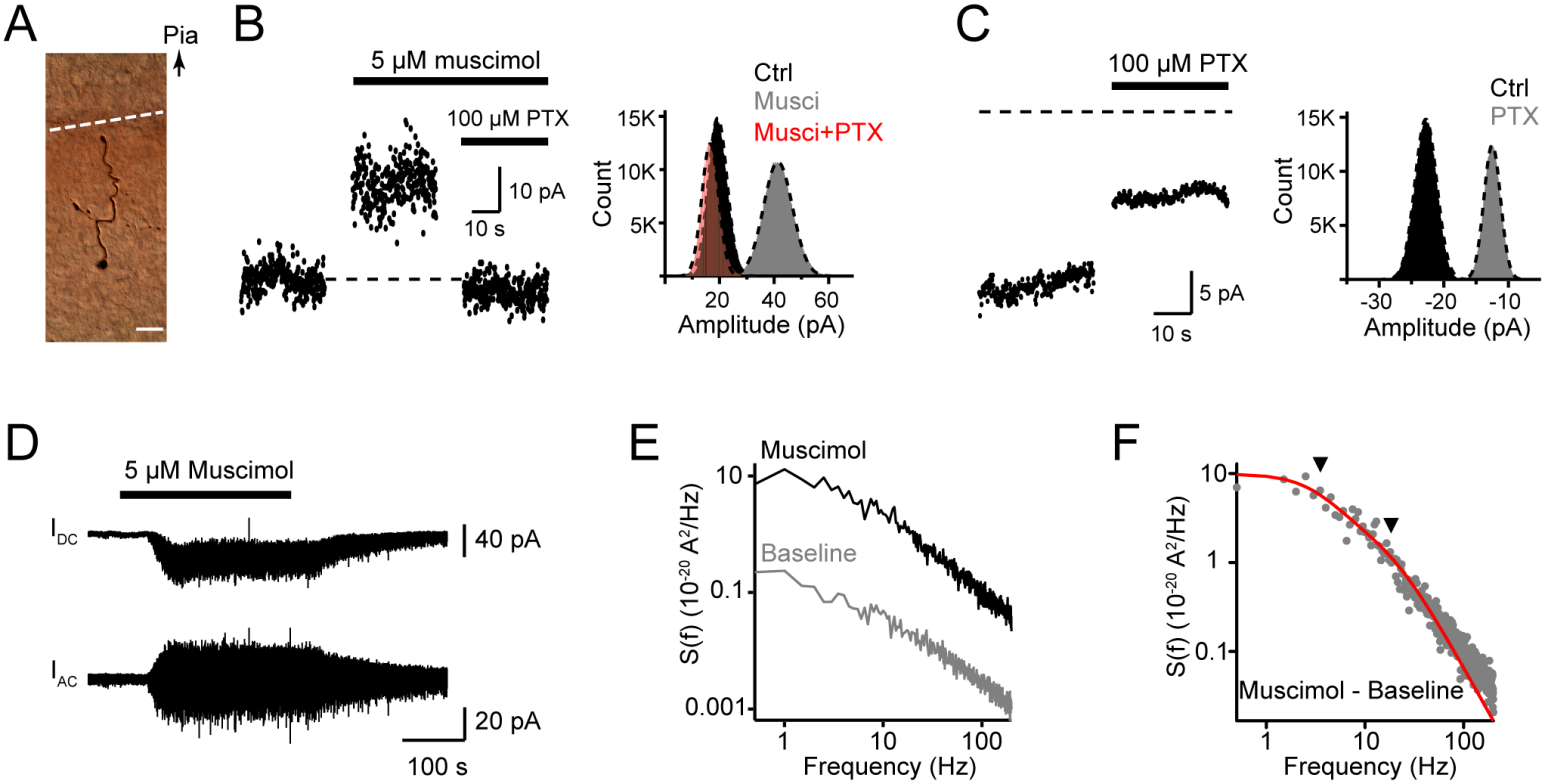
Properties of axonal GABA_A_ receptors. A, DAB staining of an isolated axon bleb. The axon bleb was mechanically isolated from the main axon trunk before recording (see the Methods section). Arrow indicates the direction of the pia. Dashed line indicates the cut. Scale bar: 20 µm. B, Left, an example trace showing an increase in outward holding current after bath application of 5 µM muscimol (V_hold_ = 10 mV) and the blockade of this increase by 100 µM PTX. The dashed line indicates the baseline holding current. The mean values of I_hold_ for “Ctrl”, “Musci” and “Musci + PTX” group were 19.8, 41.4 and 16.9 pA, respectively. Right, histograms of the membrane currents shown on the left. The best-fit curves (dashed lines) were single Gaussian distributions. Axon blebs were recorded with patch pipettes filled with a low-Cl^−^ ICS (7 mM [Cl^−^]_i_). C, Left, an example trace showing a decrease in inward holding current after bath application of 100 µM PTX (V_hold_ = –70 mV). The mean values of I_hold_ for “Ctrl” and “PTX” group were –22.8 and –12.5 pA, respectively. Right, histograms of the membrane currents shown on the left. Patch pipettes were filled with CsCl-based ICS (149 mM [Cl^−^]_i_). D, Current traces recorded in an isolated bleb. V_hold_  = –60 mV, CsCl-based ICS was used. Top, the actual current response; bottom, current trace with DC Remove. E, Power spectral density plots of membrane current fluctuations in control and muscimol-treated conditions (same data as in D). F, Subtraction of the power spectral density in the control from that with muscimol treatment (same data from D and E). The red line was the fitting curve of double Lorentzian functions. Cut-off frequencies (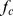) of the two components (arrowheads) were 3.5 and 18.1 Hz.

The GABA receptors at the AIS can be activated upon GABA release from the axo-axonic synapses [26], [27]. Considering the lack of direct evidence of GABAergic synapses at the axon trunk, we speculated that these axonal GABA_A_ receptors may be activated by ambient GABA. To exclude the contribution of somatodendritic GABA receptors, we performed the following experiments in isolated axon blebs that were disconnected from the soma but with axon segments attached (see the Methods section, Figure 3A). In the presence of the GABA_A_ receptor agonist muscimol (5 µM), we observed an increase in outward holding current (ΔI = 14.8±4.3 pA, n = 11; low-Cl^−^ ICS) at a holding potential of 10 mV. Again, 100 µM PTX could block this effect (Figure 3B). At a holding potential of –70 mV, we also observed a decrease in the inward holding current after the application of PTX (ΔI = –5.8±2.3 pA, n = 6; CsCl ICS), even without the presence of muscimol (Figure 3C). These results suggest that ambient GABA (mimicked by a low concentration of muscimol) can activate axonal GABA_A_ receptors.

Next, we investigated the average conductance γ and the open time constant τ of these axonal receptors by using noise analysis (see the Methods section). Membrane current responses to 5 µM muscimol were recorded at isolated blebs, and the variance 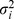 and cut-off frequency 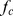 could be then obtained (Figure 3D, E, F). In most cases, the power spectral density plots of membrane current fluctuations could be well fitted by a double Lorentzian function and yielded two 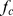 values and consequently two τ values. The γ, 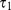, and 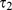 values for axonal GABA_A_ receptors activated by 5 µM muscimol were 23.4±1.6 pS, 45.7±3.0 ms and 8.2±0.9 ms, respectively (n = 10). All values were quite similar to those obtained from somatic GABA_A_ receptors by using nucleated patch recording (23.5±1.2 pS, 45.1±6.0 ms and 7.0±0.6 ms, n = 9). These results show no significant difference in single-channel properties between axonal and somatic GABA_A_ upon activation by a low concentration of muscimol.

### Axonal GABA_A_ receptors hyperpolarize the axon

Activation of GABA_A_ receptors in the somatodendritic compartments hyperpolarizes the *V*_m_ and thus inhibits and structures neuronal activities [14]. However, different results were observed for axonal GABA_A_ receptors in different subcellular locations and brain regions, such as the AIS of hippocampal [17] and neocortical pyramidal neurons [15], the axon trunk of cerebellar granule cells [19] and axon terminals of hippocampal mossy fibers [18].

**Figure 4:**
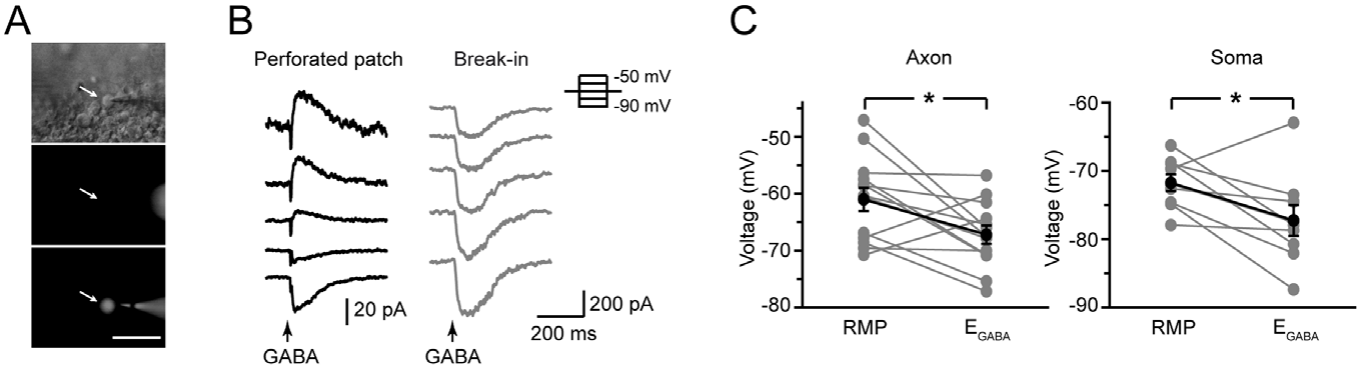
Reversal potential of GABA responses (E_GABA_) is more negative than the local RMP. A, Gramicidin perforated patch recording from an axon bleb. Arrow indicates the recorded bleb. Top, DIC image of the recording; middle, fluorescence image (unlabeled bleb); bottom, fluorescence image (labeled bleb, indicating rupture of patch membrane). Scale bar: 50 µm. B, Example traces showing GABA responses at different holding potentials (from –90 to –50 mV) before (black) and after the break-in (membrane rupture, gray). C, Comparison of E_GABA_ and RMP. Note that E_GABA_ at both the soma and the distal axon bleb were more hyperpolarized than their local RMP. *, P<0.05, paired t-test.

In our experiment, we used noninvasive gramicidin perforated patch recording to examine the reversal potential of GABA responses (E_GABA_) under physiological conditions. Because gramicidin-formed holes are not permeable to Cl^−^, the intracellular Cl^−^ gradient is not perturbed during this type of perforated patch recording. Because we added Alexa Fluor 488 to the pipette solution, rupture of the membrane would result in the presence of fluorescence in the recorded cell or bleb (Figure 4A). In these experiments, we used a high-Cl^−^ pipette solution, and rupture of the membrane would also lead to a dramatic change in E_GABA_ (Figure 4B). These results revealed that E_GABA_ at the distal axonal bleb (>100 µm apart from the soma) was –67.3±1.5 mV (n = 14), significantly more positive than that at the soma (−79.0±2.3 mV, n = 13. p<0.01, two-sample t-test). However, both values were more negative (hyperpolarizing) than the respective local resting *V*_m_ (RMP) (Figure 4C). In distal axons, the RMP and E_GABA_ were –61.0±2.1 mV and –67.2±1.6 mV (n = 13. P = 0.02, paired t-test), respectively. Similar results were obtained when we performed perforated patch recording at the soma (RMP: –71.7±1.2 mV; E_GABA_: –77.3±2.3 mV, n = 9. P = 0.03, paired t-test). Together, these results suggest that GABA functions as an inhibitory transmitter (hyperpolarizing the axon) at *V*_m_ levels more positive than the RMP.

### Axonal GABA_A_ receptors regulate AP waveform

We next investigate the role of axonal GABA_A_ receptors in regulating orthodromically propagating APs along the axon. APs were evoked by extracellular stimulation with a concentric electrode and AP waveforms were monitored at the bleb. Three types of intracellular solutions (ICS) were employed in this experiment, they were low-Cl^−^ ICS (7 mM [Cl^−^]_i_), modified ICS (20 mM [Cl^−^]_i_) and high-Cl^−^ ICS (75 mM [Cl^−^]_i_), respectively.

**Figure 5:**
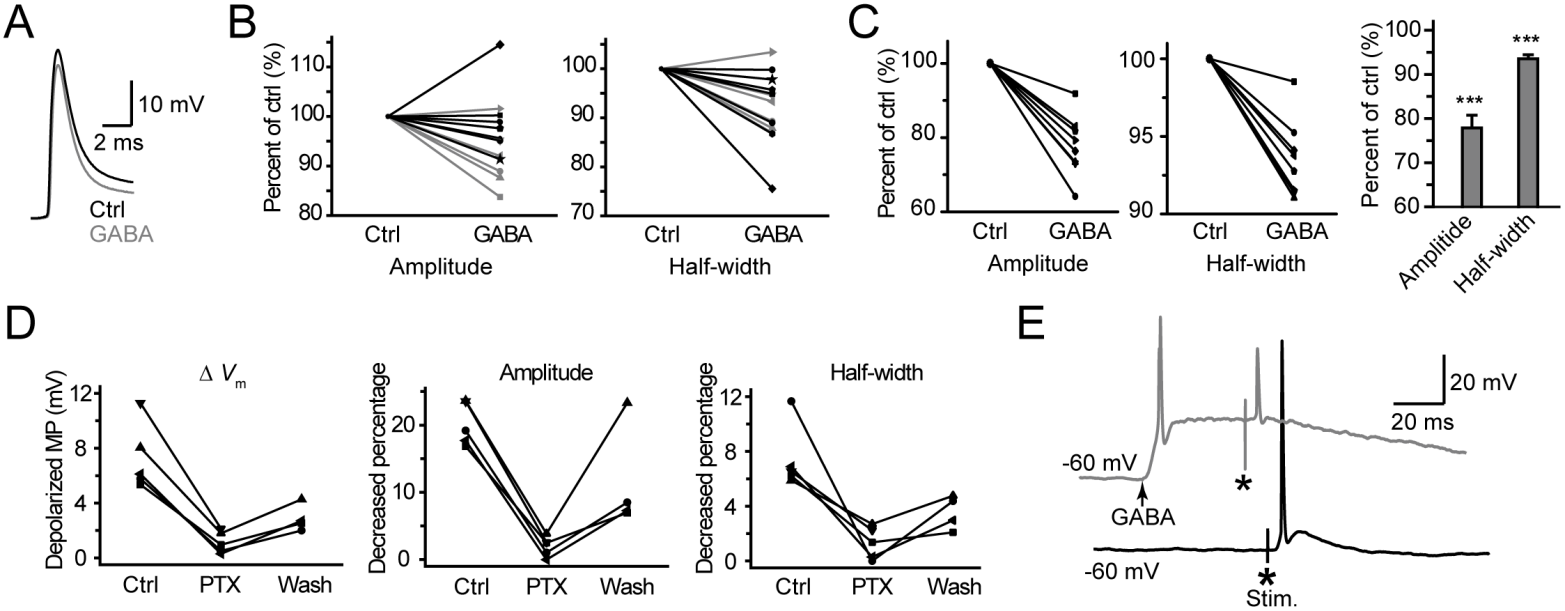
Activation of axonal GABA_A_ receptors shapes the AP waveform. A, Example traces showing the change in AP waveform after GABA iontophoresis to the axon bleb. *V*_m_ change was –3.9 mV in this bleb. The amplitude and the half-width of APs decreased to 95.6% and 86.8% of the control, respectively. B, Group data showing that activation of axonal GABA_A_ receptors shaped AP waveforms by regulating the amplitude and the half-width. Note that the recordings were performed under current-clamp and that the *V*_m_ could be manipulated by DC current injection. Black, GABA responses were hyperpolarizing; gray, depolarizing. The blebs were recorded with low-Cl^−^ ICS (7 mM [Cl^−^]_i_). C, Increasing [Cl^−^]_i_ depolarized the *V*_m_ but still showed a shunting effect on AP waveforms. Amplitude and half-width were significantly reduced. Modified ICS (20 mM [Cl^−^]_i_) was used for these recordings. ***, P<0.001, paired t-test. D, Bath application of PTX could block the GABA-induced *V*_m_ depolarization and its shunting effect on AP waveform. Modified ICS was used. E, Example traces showing that GABA application caused a shunting effect on APs evoked by electric shocks (asterisks), although GABA itself could evoke an AP (arrow). High-Cl^−^ ICS (75 mM [Cl^−^]_i_) was used here.

Based on the results that activation of axonal GABA_A_ receptors may hyperpolarize the axon, we speculated that the [Cl^−^]_i_ in the distal axon trunk should be low. We therefore used the low-Cl^−^ ICS first and found that the waveform of the evoked AP could be modulated by GABA applied to the axon bleb (Figure 5A). When the *V*_m_ was maintained at approximately –50 mV in current-clamp mode, GABA application at the bleb could cause hyperpolarization (–2.4±0.6 mV, ranging from –1.0 to –5.5 mV, n = 7). Although the amplitude showed no significant change (99.0±2.8% of control, P = 0.81, 41.3 vs. 41.0 mV, n = 7), the half-widths of APs were significantly decreased to 91.4±3.2% (P = 0.04, 1.4 vs. 1.3 ms, n = 7) of the control (black plots, Figure 5B., paired t-test). Then, we clamped the *V*_m_ of the bleb to a more hyperpolarized level at approximately –65 mV, application of GABA to the axon caused depolarization (3.4±1.1 mV, ranging from 1.0 to 8.0 mV, n = 6). Interestingly, both the AP amplitude and the AP half-width were significantly reduced (gray, Figure 5B). These values were decreased to 91.5±2.6% (P = 0.03, 78.9 vs. 72.1 mV) and 92.6±2.5% (P = 0.04, paired t-test, 0.94 vs. 0.87 ms) of the control, respectively. Together, our results indicate that GABA application at the axon reduces AP duration whether it hyperpolarizes or depolarizes the *V*_m_, presumably resulting from a shunting effect of the GABA-induced conductance.

It has been reported that axo-axonal chandelier cells innervate the AIS of postsynaptic pyramidal cells and induce depolarizing responses, due to a high [Cl^−^]_i_ at this strategic location (AP initiation site). Therefore, we increased the [Cl^−^]_i_ from 7 to 20 mM (modified ICS) to mimic conditions of relatively high [Cl^−^]_i_ at AIS, depolarizing E_GABA_ to –48.0±0.5 mV (n = 19, corrected with a liquid junction potential of 14 mV). Although GABA iontophoresis at the bleb caused a larger depolarization (8.0±0.9 mV, ranging from 5.3 to 13.6 mV, n = 8), the amplitude and the half-width of APs decreased to 77.9±2.9% and 93.5±0.9% of control (P<0.001), respectively (Figure 5C). Bath application of PTX (25–50 µM) could block these GABA-induced depolarization and AP waveform changes (Figure 5D). Again, application of baclofen at the bleb caused no significant change in either the amplitude (P = 0.18) or the half-width (P = 0.22, paired t-test. n = 5) of AP waveforms. These results further support the shunting effect of GABA_A_ receptors on AP waveform at the axon.

Further increasing the [Cl^−^]_i_ in the pipette solution to 75 mM (high-Cl^−^ ICS; reversal potential: –10.8±0.6 mV, corrected with a liquid junction potential of 9 mV, n = 17) produced larger depolarization and even facilitated the generation of APs. As shown in Figure 5E, GABA iontophoresis could evoke a large depolarization and sometimes even generate APs (arrow). Again, this GABA response also showed an inhibitory effect on the AP waveform when the orthodromic AP was evoked during GABA application (asterisk, Figure 5E). The dramatic decrease in AP amplitude resulted from an increase in membrane conductance (shunting effect) and a decrease in Na^+^ channel availability (depolarization-induced inactivation). Interestingly, GABA-evoked depolarization could not promote the initiation of APs even when the strength of extracellular stimulation was very close to the threshold (data not shown).

Together, these results indicate that the waveforms of orthodromically propagating APs at the axon can be modulated by axonal GABA_A_ receptors. Activation of these receptors mainly showed a shunting effect on AP waveforms regardless of the polarity of GABA-induced *V*_m_ changes.

### Propagation of GABA-induced hyperpolarization along the axon

**Figure 6:**
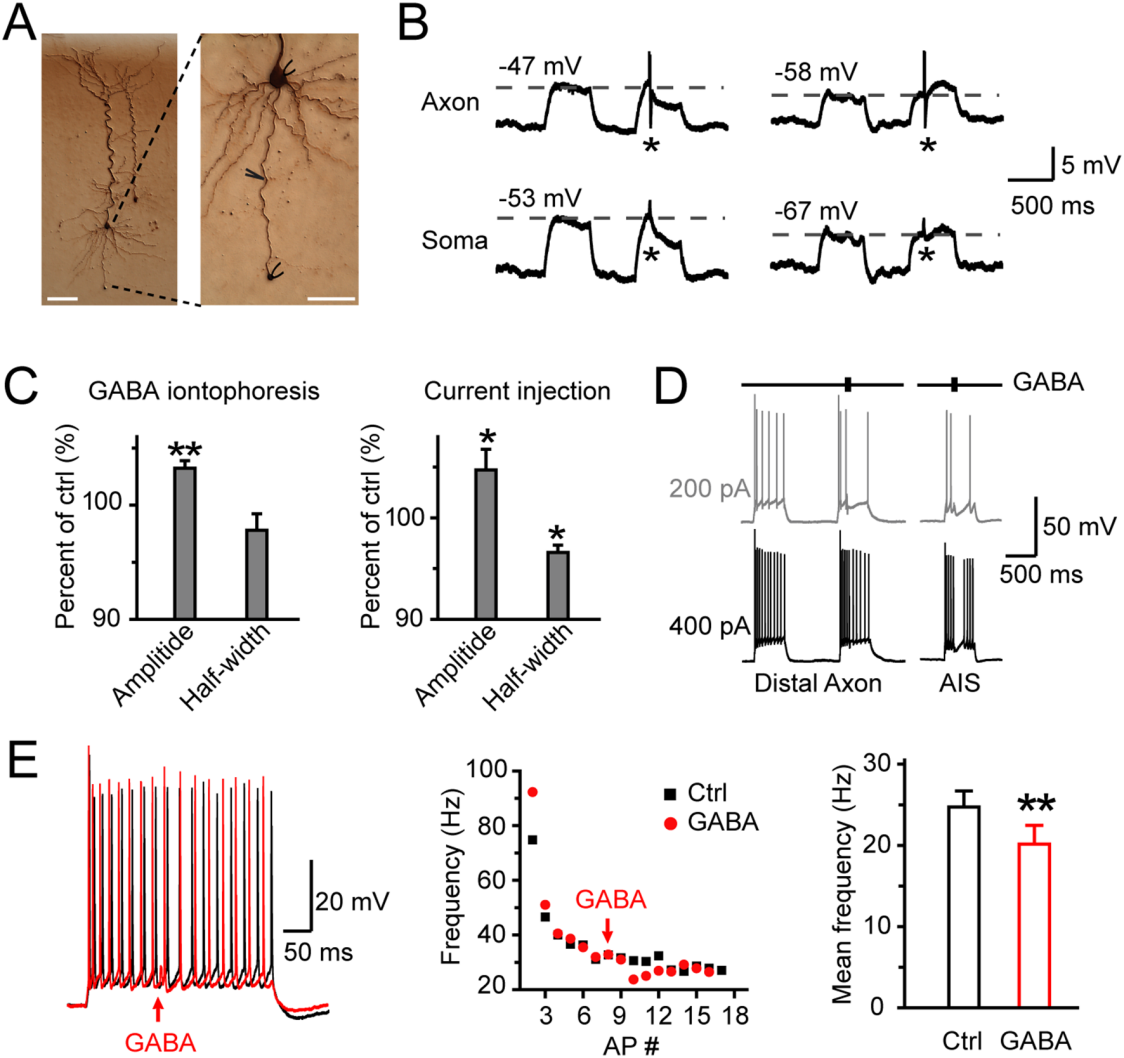
Propagation of GABA-induced hyperpolarization at the axon regulates AP generation. A, DAB staining of recorded neurons. Simultaneous recording from the soma and axon bleb were performed in a pyramidal neuron (left), and GABA was applied to the axon trunk (right). The axon length was 239 µm in this case. The distance between the iontophoresis site and the soma was 117 µm. Scale bar: 100 µm (left); 50 µm (right). B, The sign of the effect of GABA (hyperpolarization or depolarization) depended on the *V*_m_. Top, traces were taken from the bleb. Bottom, traces were the corresponding responses at the soma. The *V_m_* was clamped through somatic DC current injection. Asterisk indicates application of GABA to the main trunk. C, Left, application of GABA to the axon increased the amplitude but decreased the half-width of propagating APs. GABA iontophoresis hyperpolarized the *V*_m_ by 2.3±0.4 mV (n = 7). Right, similar results were obtained when *V*_m_ was hyperpolarized by 2.8±0.3 mV (n = 5) through DC current injection. *, P<0.05; **, P<0.01, paired t-test. D, Example traces showing activation of axonal GABA_A_ receptors reduced firing probability and frequency. The distances between the iontophoresis site and the soma were 100 µm (distal axon) and 18 µm (AIS). E, Left, repetitive firing recorded at an axon bleb induced by 400 pA DC current injection at the soma before (black) and after (red) GABA application to the axon trunk. The arrow indicates GABA iontophoresis. Middle, instantaneous firing frequency of APs decreased after GABA application (same data as shown in the left). Right, group data showing a decrease in the mean frequency of APs after GABA iontophoresis at the axon trunk. **, P<0.01, paired t-test. Low-Cl^−^ ICS was used in these experiments.

The shunting effect induced by increased conductance may occur only at the location where the receptors were activated; however, it remains unclear whether GABA-induced *V*_m_ changes at remote locations could influence AP initiation and propagation. To address this question, we performed simultaneous recordings from the soma and the bleb with low-Cl^−^ ICS, and then applied GABA through iontophoresis to the axon trunk to investigate its role in regulating AP generation (Figure 6A).

GABA application to the axon trunk could result in *V*_m_ hyperpolarization or depolarization (with low-Cl^−^ ICS), depending on the *V*_m_ when GABA was applied. These *V*_m_ changes could propagate to the soma and the recorded bleb (Figure 6B). Because E_GABA_ at the axon was more negative than the RMP (Figure 4), GABA receptor activation could hyperpolarize the axon. Given that the voltage fluctuations could propagate along the main axon with a length constant of hundreds of micrometers [2], *V*_m_ hyperpolarization could spread along the axon trunk and even reach the presynaptic terminals. Activation of axonal GABA receptors at the main trunk could increase the amplitude (by 3.2±0.7%, n = 7. P<0.01) but did not produce a significant change in AP half-width (decreased by 2.2±1.4%, n = 7. P = 0.15). This phenomenon was similar to the AP waveform changes caused by hyperpolarization induced by negative current injection (amplitude: by 4.7±2.0%, P = 0.04; half-width: by 3.4±0.7%, P = 0.04; n = 5. Figure 6C). Therefore, activation of remote axonal GABA receptors could also regulate the waveform of propagating APs by hyperpolarizing the membrane potential.

We next injected step currents (100–500 pA, 500 ms) at the soma to evoke a train of APs, and corresponding APs could be observed at the bleb. As shown in Figure 6D, we observed a failure of AP initiation for weak somatic stimulation (gray, 100–200 pA, n = 3) and a decrease in firing frequency for strong stimulation (black, 400–500 pA, n = 5) when GABA was applied to the distal axon trunk (>100 µm apart from the soma). In contrast, when GABA was delivered at the AIS, substantial hyperpolarization (recording with pipettes filled with Low-Cl^−^ ICS) and failure of AP initiation could be observed during both weak and strong current stimulation (200 and 400 pA, n = 7), presumably due to the strategic function of AIS for AP initiation. Iontophoresis of GABA to the main trunk could cause a decrease in the instantaneous firing frequency at the distal axon bleb (Figure 6E). The mean frequency of APs evoked by step current injection (400–500 pA) dropped from 24.7±2.0 to 20.1±2.3 Hz (P<0.01, paired t-test. n = 5) immediately after GABA application (Figure 6E). These results indicate a role for axonal GABA_A_ receptors in regulating AP generation and firing frequency.

### Regulation of AP-induced Ca^2+^ transients by axonal GABA_A_ receptors

Previous studies revealed that changes in AP waveforms could regulate the amount of Ca^2+^ entry, leading to changes in neurotransmitter release [28], [29]. We therefore speculated that AP waveform changes induced by the activation of axonal GABA_A_ receptors could also regulate intracellular Ca^2+^ levels in the axon and the consequent neurotransmitter release.

**Figure 7:**
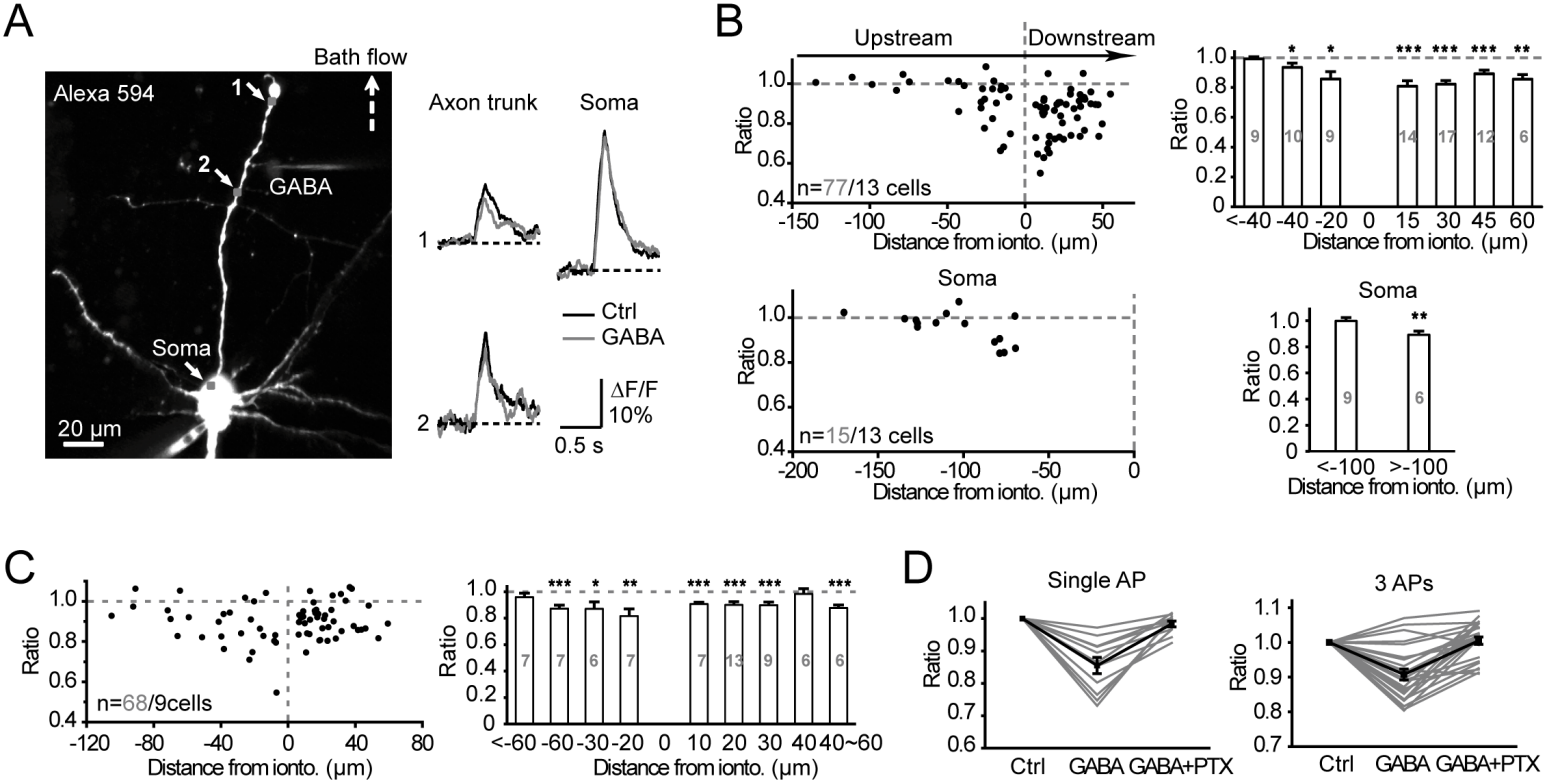
Activation of axonal GABA_A_ receptors decreases AP-induced Ca^2+^ transients. A, Left, projection of 2-photon fluorescence images of a recorded pyramidal neuron filled with Alexa Fluor 594 (50 µM) and OGB-1 (200 µM). The dashed arrow indicates the direction of bath flow. Right, iontophoresis of GABA at the axon trunk (labeled by “GABA” in the left image) decreased Ca^2+^ transients evoked by single APs at nearby locations (ROI 1 and 2) but showed no effect on somatic transients. B, Group data showing the effect of GABA on single-AP-triggered Ca^2+^ transients. The results are presented as the ratio of the Ca^2+^ signal amplitude for “GABA” to “Ctrl.” Top, data collected from the axon trunk (n = 13 axons). Bottom, data collected from the soma. The site for GABA iontophoresis was defined as 0. If the ROI was located downstream from the iontophoresis site, the sign of the distance was positive; otherwise it was assigned a negative sign. *, P<0.05; **, P<0.01; ***, P<0.001, paired t-test. C, Group data showing the effect of GABA on Ca^2+^ transients induced by 3 APs (100 Hz). Data were collected from the axon trunk (n = 9 axons). D, PTX blocked GABA-induced decrease in Ca^2+^ transients. Gray, individual cells; black, average data.

We performed two-photon Ca^2+^ imaging to investigate whether AP-induced Ca^2+^ transients at the axon were subject to modulation by iontophoresis of GABA. As shown in Figure 7A, a layer –5 pyramidal neuron was filled with Alexa Fluor 594 (50 µM) and the Ca^2+^ indicator OGB-1 (200 µM) through the whole-cell patch pipette. Several regions of interest (ROIs) were selected for optical recording. We observed a decrease in Ca^2+^ transients in the axon (ROI 1 and 2, Figure 7A) after GABA application; however, the Ca^2+^ signals at the soma, which was remote from the GABA iontophoresis site, were not affected. In this particular cell, the distance between the iontophoresis site and ROI 1, ROI 2 and the soma were 36, 21 and 127 µm, respectively. The direction of bath flow was always from the soma to the axon, and the GABA application site was defined as 0. The distances between the application site and downstream or upstream ROIs were shown as positive or negative values, respectively. Group data from 13 cells showed that local GABA application decreased the Ca^2+^ transients induced by single APs by 14.1±1.4% at the axonal segments (ranging from –39 to 55 µm, n = 68 ROIs) that were close to the iontophoresis site (Figure 7B); no significant change was observed in 9 ROIs with distances longer than –40 µm. In contrast, Ca^2+^ transients at the soma showed no significant decrease (P = 0.84) after GABA application at a distance>100 µm from the soma. Significant reduction (P<0.01) in Ca^2+^ transients was detected only when the distance between the soma and GABA application site was less than 100 µm (Figure 7B).

Similarly, axonal Ca^2+^ transients evoked by a train of 3 APs at 100 Hz were also significantly decreased by local GABA application (by 10.9±1.2%, axonal segments ranging from –59 to 59 µm, n = 61 ROIs of 9 cells. Figure 7C); no significant change was observed in 7 ROIs with distance greater than –60 µm. Bath application of PTX could block the GABA-induced decrease in Ca^2+^ transients evoked by either single APs (n = 11 ROIs of 3 cells) or bursts of 3 APs (n = 25 ROIs of 6 cells), indicating a role for axonal GABA_A_ receptors in modulating local Ca^2+^ signals (Figure 7D).

## Discussion

In this study, we found that GABA_A_ receptors were expressed at the main axon trunks of layer – 5 pyramidal neurons and that activation of these receptors mainly hyperpolarized local *V*_m_ under physiological conditions. Importantly, we also show that waveforms of propagating APs could be shaped by either GABA-induced shunting or hyperpolarizing effects. Consistent with these results, AP-induced Ca^2+^ transients were substantially reduced by GABA application to the axon trunk, suggesting an important role for axonal GABA_A_ receptors in regulating the output signal of pyramidal neurons.

Previous studies revealed the expression of GABA_A_ receptors at presynaptic axons [24], [30], [31], [32], [33], [34]. Those axonal GABA_A_ receptors have been described as localizing in various brain regions including the spinal cord, brainstem, the cerebellum, the hippocampus and the cortex. Due to difficulties in recording from thin axons with diameter less than 1 µm [35], the properties and function of axonal GABA receptors have received much less attention than somatodendritic receptors. At the axon, GABA receptors distributed in some special axonal compartments such as the AIS (thicker than the main axon) and axon terminals (e.g., hippocampal mossy fiber boutons and midbrain calyx of Held) have been studied. In this study, by using the bleb recording method [2], we could directly examine the properties of ion channels and transmitter receptors located in the axon trunk. A previous study using high-Cl^−^ ICS (approximately 140 mM) revealed that pairing GABA iontophoresis with antidromic axonal stimulation could substantially decrease spike failures [36]. Here, we employed multiple types of ICS with varying Cl^−^ concentrations and showed the existence of GABA_A_ receptors at the distal axon of layer –5 pyramidal neuron in prefrontal cortex. In addition, both that previous study and our findings revealed no presence of glutamate receptors at the axon, which is different from the pattern found in CA3 pyramidal neurons [7].

There is still debate regarding whether activation of axonal GABA_A_ receptors induces hyperpolarization or depolarization. To address this question, one must know the difference between the RMP and E_GABA_ at the axon. With a variety of noninvasive recording methods performed on axon including the AIS [15], [37], axon trunk [19] and presynaptic terminals [18], previous studies showed that axonal E_GABA_ was more depolarized than RMP so GABA responses increased neuron excitability. However, the unitary field potential recordings performed on hippocampal CA1 pyramidal cells suggested that activation of axo-axonic synapses induces hyperpolarizing responses [17]. In our experiments, we found that E_GABA_ in the distal axon trunk (beyond the AIS) is more hyperpolarized than the local RMP (–67 vs. –61 mV). Unlike some of the reports mentioned above, our findings suggest that GABA can induce hyperpolarization at distal axonal segments. Because E_GABA_ is mainly determined by [Cl^−^]_i_, the conflicting results could be explained by different [Cl^−^]_i_. A higher [Cl^−^]_i_ can result in a more depolarized E_GABA_. The [Cl^−^]_i_ varies across developmental stages and brain regions. Even within the same brain region, it has also been reported that the response induced by GABA can be cell-type specific [38], [39], [40]. At one extreme, the GABA-mediated response in the suprachiasmatic nucleus is excitatory during the day but inhibitory during the night [41]. Various cation-chloride co-transporters play pivotal roles in maintaining intracellular chloride homeostasis [42], [43]. The Na^+^-K^+^-2Cl^−^ co-transporters (NKCCs) account for Cl^−^ accumulation, whereas K^+^-2Cl^−^ co-transporters (KCCs) account for Cl^−^ extrusion. In retina, the expression pattern of cation-chloride co-transporters shows specificity in cell type and subcellular locations [44]. In cortex, the co-operation of NKCC1 and KCC2 results in a relatively high [Cl^−^]_i_ in AIS and therefore depolarizes E_GABA_ [15], [37]. The distribution pattern and function of these co-transporters at the axon trunk remains to be further examined.

However, hyperpolarization and depolarization cannot simply be considered as inhibitory and excitatory, respectively. Under some circumstances, presynaptic depolarization mediated by axonal GABA_A_ receptors results in activation of voltage-gated Ca^2+^ channels, even causing AP initiation [45]. In other cases, presynaptic inhibition occurs because of inactivation of Na^+^ and/or Ca^2+^ channels. GABA-induced depolarization may reduce the amplitude of APs and cause a decrease in Ca^2+^ influx [24], [46]. Furthermore, opening of GABA_A_ receptor channels will introduce a leaky conductance during generation of APs and propagation of postsynaptic potentials whether the GABA response is depolarizing or hyperpolarizing [47], [48]. The actual effect of GABA also depends on the precision of the timing and location [49], [50]. Therefore, the shunting inhibition together with *V*_m_ changes must be taken into account in analyses of the effect of GABA. In our study, GABA-mediated inhibition is associated with both hyperpolarization and shunting under physiological conditions. By increasing the [Cl^−^]_i_ to a high level (75 mM), GABA application itself can evoke APs if the depolarizing response overcomes the shunting conductance and reaches the threshold for AP generation (Fig. 5E).

The concentration of GABA in the synaptic cleft can reach a millimolar level [51], [52] and the ambient GABA concentration varies from nanomolar to a few micromolar [53], [54]. GABA_A_ receptors located in the distal axon trunk can be activated by a low concentration of muscimol (5 µM). Additionally, the change of holding current in the presence of PTX was consistent with previous findings [55], [56]. These results suggest that axonal GABA_A_ receptors have high affinity to GABA and can be tonically activated. Before entering the white matter, the axon segments (approximately 400 µm in length) of layer –5 pyramidal neurons in prefrontal cortex are not myelinated [57]. These axon segments may be subcellular candidates for the modulation of neuronal output signals. We have recently shown that at these unmyelinated axons K^+^ currents and AP waveforms are subject to dopaminergic modulation [58]. At the direct activation site, the GABA-induced shunting effect can regulate the AP waveform by decreasing the amplitude and the half-width. Beyond this site, GABA-induced hyperpolarization can propagate for hundreds of micrometers and decrease the AP width in contrast to the action of subthreshold depolarization [2], [59]. Consistent with previous findings showing a linear dependence of Ca^2+^ influx on AP half-width [28], [60], our Ca^2+^ imaging results show a decrease in AP-induced Ca^2+^ transients after GABA application to the axon.

In summary, we show the existence of GABA_A_ receptors at the axon trunk of cortical pyramidal neurons. Activation of these receptors shortens propagating APs and decrease AP-induced Ca^2+^ entry by shunting and/or hyperpolarization. GABA-induced changes in axonal AP waveforms may represent a new mechanism for GABAergic modulation of synaptic transmission in the cortex.

## Supporting Information

Figure S1 **Filtering effect of the axon cable on GABA-induced currents.** A, Example recording at the soma with GABA iontophoresis at the axon bleb. The red line indicates the linear fit of the rising phase, the rising slope can be derived from this fit. The red dashed line is an exponential fit of the decay phase, the decay time course can be then obtained. In this experiment, whole-cell recording was achieved at the soma with low-Cl^−^ ICS (V_hold_ = –40 mV) while GABA was applied at the axon bleb via iontophoresis (200 nA, 5 ms). B, Left, a plot of the rising slope of the GABA-induced currents as a function of the distance between the bleb and the soma (distance *L*, n = 29). Right, the pooled data shown on the left were divided into three subgroups according to the distance *L*. The rising slope of each group was 6.0±0.7 (n = 6), 2.0±0.4 (n = 15), 0.9±0.3 (n = 8), respectively. C, Left, a plot of the decay time course as a function of the distance *L* (n = 29). Right, the decay time course of each subgroup was 168.4±47.6 (n = 6), 216.6±34.1 (n = 15), 305.8±88.2 (n = 8), respectively.
(TIF)
