# Supplementary figures and images for: Regulation of Action Potential Waveforms by Axonal GABAA Receptors in Cortical Pyramidal Neurons
Source: PLoS One. 2014 Jun 27;9(6):e100968. doi: 10.1371/journal.pone.0100968 (PMC4074163; doi:10.1371/journal.pone.0100968)

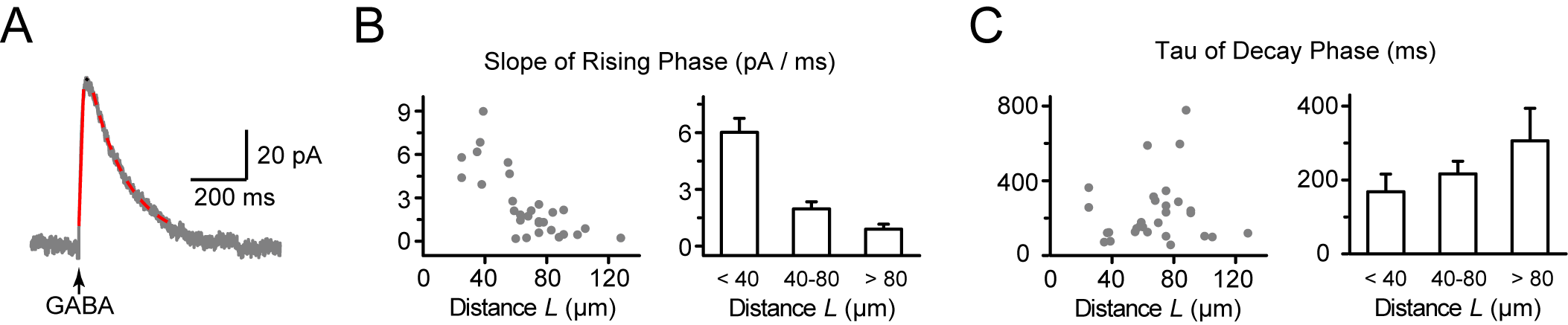

Supplement: Figure S1 — Filtering effect of the axon cable on GABA-induced currents. A, Example recording at the soma with GABA iontophoresis at the axon bleb. The red line indicates the linear fit of the rising phase, the rising slope can be derived from this fit. The red dashed line is an exponential fit of the decay phase, the decay time course can be then obtained. In this experiment, whole-cell recording was achieved at the soma with low-Cl− ICS (Vhold = –40 mV) while GABA was applied at the axon bleb via iontophoresis (200 nA, 5 ms). B, Left, a plot of the rising slope of the GABA-induced currents as a function of the distance between the bleb and the soma (distance L, n = 29). Right, the pooled data shown on the left were divided into three subgroups according to the distance L. The rising slope of each group was 6.0±0.7 (n = 6), 2.0±0.4 (n = 15), 0.9±0.3 (n = 8), respectively. C, Left, a plot of the decay time course as a function of the distance L (n = 29). Right, the decay time course of each subgroup was 168.4±47.6 (n = 6), 216.6±34.1 (n = 15), 305.8±88.2 (n = 8), respectively. (TIF) [file pone.0100968.s001.tif]
